# Neuroprotective Effect of Resveratrol Propionate Esters on Apoptosis of SH-SY5Y Cells Induced by Hydrogen Peroxide

**DOI:** 10.1155/bri/9973711

**Published:** 2025-09-10

**Authors:** Chun-Yung Huang, Chih-Yao Hou, Shin-Yu Chen, Chang-Wei Hsieh, You-Lin Tain, Mei-Chun Lin, Yu-Wei Chen, Ming-Kuei Shih

**Affiliations:** ^1^Department of Seafood Science, College of Hydrosphere, National Kaohsiung University of Science and Technology, Kaohsiung 81157, Taiwan; ^2^Department of Food Science, National Pingtung University of Science and Technology, Pingtung 91201, Taiwan; ^3^Department of Food Science and Biotechnology, National Chung Hsing University, Taichung 40227, Taiwan; ^4^Department of Medical Research, China Medical University Hospital, Taichung 40402, Taiwan; ^5^Department of Food Science, National Ilan University, Yilan 260007, Taiwan; ^6^Department of Pediatrics, Kaohsiung Chang Gung Memorial Hospital, Kaohsiung 83301, Taiwan; ^7^Institute for Translational Research in Biomedicine, Kaohsiung Chang Gung Memorial Hospital, Kaohsiung 83301, Taiwan; ^8^College of Medicine, Chang Gung University, Taoyuan 33305, Taiwan; ^9^Graduate Institute of Food Culture and Innovation, National Kaohsiung University of Hospitality and Tourism, Kaohsiung 81271, Taiwan

**Keywords:** apoptosis, flow cytometry, neuroprotection, propionate resveratrol, SH-SY5Y cells

## Abstract

Resveratrol, often referred to as 3,4′,5-trihydroxystilbene (RSV), is a compound with a variety of pharmacological benefits, such as antiaging, neuroprotective, chemopreventive, antioxidant, and anti-inflammatory qualities. To investigate neuroprotective properties of resveratrol propionate (RPE), H_2_O_2_ was added to SH-SY5Y cells (human neuroblastoma cell line) that had been pretreated with RPE. The modulation of Bcl-2 and Bax proteins, the mitochondrial membrane potential (MMP), cytochrome c release, the activation of caspase-9 and caspase-3, DNA fragmentation, and membrane catenin analysis were all measured in this study using flow cytometry. The protective effect of RPE pretreatment of SH-SY5Y cells on the H_2_O_2_-induced death process was further investigated using annexin V-fluorescein isothiocyanate (FITC). The induction of caspase-9 and caspase-3, release of cytochrome c, loss of MMP, modification of Bcl-2 and Bax, DNA fragmentation, and annexin V-FITC/PI (propidium iodide) double staining all indicate that RPEs are capable of successfully shielding SH-SY5Y cells from H_2_O_2_-induced cytotoxicity. Therefore, RPE is considered a good candidate for the prevention and treatment of neurodegeneration induced by oxidative damage.

## 1. Introduction

The incidence of neurodegenerative disorders, such as Alzheimer's disease (AD) and Parkinson's disease (PD), is on the rise due to the advancing age of the population. There is evidence that oxidative damage can cause problems for the nervous system and brain. There is much evidence that oxidative stress damages cells and plays a part in both the normal aging process and the development of neurodegenerative diseases like AD and PD [1–3]. Therefore, a therapeutic approach could be devised by augmenting or fortifying the innate mechanism of protection against oxidative stress through the consumption of antioxidants in the diet or the administration of pharmacological agents. Consequently, it is critical to find substances that, while limiting severe side effects, can neutralize the effects of free radicals and shield neural cells from oxidative damage.

Multiple biological activities are associated with resveratrol (3,4′,5-trihydroxystilbene, RSV), including antioxidative [4], anti-inflammatory [5], chemopreventive [6], neuroprotective [7], antiobesity [8], and antiaging [9]. Studies on the inhibition of oxidative damage by resveratrol or polyphenols and the protection of neurons through SH-SY5Y cells [10–16] also echo the fact that oxidative damage is highly relevant to neurodegenerative diseases [17]. The positive therapeutic potential of RSV in human neurodegenerative diseases such as PD [18–20] and AD [21–23] is particularly noteworthy. However, RSV has poor pharmacokinetic and pharmacodynamic properties, and human and animal experimental models have shown low bioavailability [24–27], severely restricting its use. Our previous study demonstrated that N-ethyl-N′-(3-dimethylaminopropyl)carbodiimide (EDC) and 4-dimethyl aminopyridine (DMAP) successfully improved the Steglich esterification reaction [28] to produce short-chain fatty acid resveratrol esters, including resveratrol acetylate esters (RAE), resveratrol propionate esters (RPEs), and resveratrol butyrate esters (RBEs). Inhibiting β-carotene bleaching and Cu^2+^-induced oxidation of low-density lipoprotein (LDL) was the most prominent effect of RPE [29]. Our prior research documented the synthesis of RBE through the esterification of butyric acid and RSV in an effort to increase the poor bioavailability of the original RSV [30]. We evaluated the antioxidant capacity, lipid biosynthesis, candidate gene regulation, liver protection ability, obesity inhibition, and intestinal bacteria adjustment effects of RBE in vitro [29], in a cell model [31], and in animal experiments [32, 33]. It has been determined that RBE complexes possess substantial antioxidant and antifat accumulation capabilities. The isolation, identification, and bioactive properties of RBE derivatives, which are crucial for their practical application as therapeutic agents [34], were first documented by Shih et al. (2021). Recently, Arbo et al. reported a series of studies on RSV modification in PD treatment, indicating that structural changes in RSV molecules, including hydroxylation, amination/amidation/immination, methoxylation, prenylation, and glycosylation, may lead to the development of derivatives with enhanced bioavailability and pharmacological activity [35]. As RPEs are novel synthetic compounds, this study expands on our previous findings [29] to facilitate the widespread application of RSV and enhance its biological activity. The potential of RBE and their separated and purified components to shield HepG2 from hydrogen peroxide (H_2_O_2_)-induced intracellular ROS has been demonstrated in our earlier investigation [34]. Recent reviews have highlighted that while SH-SY5Y cells are widely used in PD research due to their human origin and catecholaminergic characteristics, they are not strictly dopaminergic, and their suitability as a PD model depends heavily on specific differentiation protocols and culture conditions [36]. This finding underscores the importance of clarifying the limitations and appropriate applications of this cell line in neurodegenerative studies. In this study, we focused on the antioxidant and cytoprotective effects of RPE against oxidative stress-induced neuronal damage using SH-SY5Y cells as an in vitro neuroblastoma-derived model. Although these cells are widely utilized in neurotoxicity and neuroprotection studies, they are not inherently dopaminergic without appropriate differentiation. Our aim was not to model PD pathology directly, but rather to examine the general neuroprotective potential of RPE in preventing H_2_O_2_-induced apoptosis. To our knowledge, no prior studies have specifically investigated the ability of RPE to reverse oxidative stress-mediated cytotoxicity in undifferentiated SH-SY5Y cells.

## 2. Materials and Methods

### 2.1. Materials and Chemicals

Sodium carbonate, potassium sulfate, H_2_O_2_, 3-(4,5-dimethylthiazol-2-yl)-2,5-diphenyltetrazolium bromide (MTT), bovine serum albumin (BSA), Bradford reagent, and dimethyl sulfoxide (DMSO) were obtained from Sigma-Aldrich (St. Louis, MO, USA). Potassium persulfate, potassium bromide (KBr), and sodium sulfite were purchased from Merck (Darmstadt, Germany). Fetal bovine serum (FBS), trypsin/EDTA, penicillin, and streptomycin were obtained from Gibco Laboratories (Grand Island, NY, USA). Dulbecco's modification of Eagle's medium/Ham's F-12 50/50 Mix medium was obtained from Corning (Corning, NY, USA). All other reagents were of analytical grade or the best grade available.

### 2.2. Cell Culture

Food Industry Research and Development Institute provided the SH-SY5Y human dopaminergic, neuroblastoma (ATCC CRL-2266) cell line. At 37°C, with 95% air and 5% CO_2_, SH-SY5Y cells were cultured in DMEM/F-12 Mix (Dulbecco's modified Eagle's medium/Nutrient Mixture F-12m, 50/50) medium supplemented with 10% FBS, 100 units penicillin, and 100 μg/mL streptomycin. The medium was replaced at intervals of 48–72 h. For all assays, cells were seeded in tissue culture-treated plates (96-well or 6 cm; Nunc, Thermo Fisher Scientific) without any surface coating.

### 2.3. Cell Viability Test

A quantitative colorimetric 3-(4,5-dimethylthiazol-2-yl)-2,5-diphenyltetrazolium bromide (MTT) assay was used to measure cell viability [37, 38]. In summary, a culture medium was used to seed 1 × 10^5^/mL of SH-SY5Y cells in a 96-well plate (Nunc, Thermo Fisher Scientific), and the cells were then incubated for 24 h at 37°C with 5% CO_2_ in a humidified atmosphere before treatment. Cells were treated with test compounds at various concentrations and durations, and serum-free medium was used only during the H_2_O_2_ treatment step to avoid interference from serum components. After removing the treatment media and adding MTT reagent (final concentration 0.5 mg/mL), the reaction was stopped at 37°C in 5% CO_2_ after 2 h. MTT was removed, and cells were lysed with DMSO. An ELISA plate reader (PowerWave 340, Bio-Tek Instruments, Winooski, Vermont, USA) was used to measure the absorbance at 570 nm. The following formula was used to get cell viability (%):

(1) $$\begin{array}{c} \text{Cell viability}\left(\%\right)=\left(\frac{T}{C}\right)\times 100, \end{array}$$

where *C* represents the absorbance of the control and *T* is the absorbance of the test sample.

### 2.4. Flow Cytometry-Based Analyses

In all flow cytometry-based analyses, cells (4 × 10^4^ cells/mL) were seeded in 6-cm dishes (Nunc, Thermo Fisher Scientific) without surface coating and incubated with or without 2.5 or 5 μM RPE for 24 h. Following the pretreatment, cells were exposed to 2 mM H_2_O_2_ in serum-free medium for 2 h. The H_2_O_2_ solution was freshly diluted in PBS immediately prior to each experiment and used within 15 min to ensure reactivity and minimize degradation. After treatment, the cells were detached using trypsin, rinsed twice with cold PBS, and collected for analysis. All flow cytometry assays were conducted in accordance with the protocols described below.

#### 2.4.1. Mitochondrial Membrane Potential (MMP) Analysis [39]

SH-SY5Y cells were plated at a density of 1 × 10^6^ cells/mL in 24-well plates containing DMEM/F-12 (50/50) growth medium and incubated for 24 h at 37°C in a humidified atmosphere with 5% CO_2_. Cells were then pretreated with RPE at various concentrations for 24 h, followed by exposure to H_2_O_2_ (final concentration: 2 mM) for 2 h. Floating and adherent cells were collected and washed twice with 1x ice-cold PBS. The resulting SH-SY5Y cell pellets were resuspended to a final density of 1 × 10^6^ cells/mL in staining buffer and incubated at 37°C for 15–30 min in the dark.

To evaluate MMP, cells were stained with tetramethylrhodamine ethyl ester perchlorate (TMRE), obtained from Molecular Probes, Invitrogen Corp. (Catalog No. T669, Carlsbad, CA, USA), at a final working concentration of 100 nM, according to the manufacturer's instructions. TMRE was removed by centrifugation, and cells were subsequently washed twice and resuspended in fresh staining buffer for flow cytometric analysis. A minimum of 10,000 cells per sample were acquired using a BD Accuri C6 flow cytometer (BD Biosciences, San Jose, CA, USA). Data acquisition and analysis were performed using the BD Accuri C6 software. A representative scatter plot for this analysis is provided in Figure S1 (Supporting Information).

#### 2.4.2. Bcl-2 and Bax Expression Analyses [40]

Single-cell suspensions were fixed in fixation buffer at 37°C for 20 min to evaluate the expression of apoptosis-related proteins. After permeabilization with permeabilization buffer, the cells were incubated in the dark for 1 h at room temperature (20°C–22°C) with a FITC-conjugated anti-human Bcl-2 monoclonal antibody (Sino Biological, Catalog No. 100126-R204-F, Beijing, China) at a dilution of 1:100. For Bax detection, a similar procedure was followed. Cells were fixed and permeabilized as described above, then incubated with a FITC-conjugated anti-Bax antibody (BioLegend, Catalog No. #633901, San Diego, CA, USA) at a dilution of 1:100. Following staining, all samples were washed and resuspended in staining buffer prior to flow cytometry analysis. A representative scatter plot for this analysis is provided in Figures S2 and S3 (Supporting Information).

#### 2.4.3. Cytochrome c Release Analysis [38]

Cytochrome c is released from mitochondria into the cytosol during the process of apoptosis, where it contributes to the activation of caspases, a family of proteases involved in cell death. This release is considered a kinetically invariant and rapid event. In this study, single-cell suspensions were fixed using fixation buffer for 20 min at 37°C. After permeabilization with permeabilization buffer, the SH-SY5Y cells were incubated for 1 h at room temperature (20°C–22°C) in the dark with a FITC-conjugated anti-human cytochrome c monoclonal antibody (Clone 6H2, eBioscience, Catalog No. 14-6601-82, Thermo Fisher Scientific, Waltham, MA, USA) at a dilution of 1:50. Cells were subsequently washed and resuspended in staining buffer for flow cytometric analysis. A representative scatter plot for this analysis is provided in Figure S4 (Supporting Information).

#### 2.4.4. Activated Caspase-9 and Caspase-3 Analyses [38]

The single-cell suspensions were incubated in the dark at 37°C for 1 h using staining solutions containing fluorescein-conjugated inhibitors specific to caspase-9 and caspase-3 to evaluate their activation levels. For caspase-9 activity, the CaspGLOW Fluorescein Active Caspase-9 Staining Kit (Catalog No. 88-7006, Thermo Fisher Scientific, Waltham, MA, USA) was used at a dilution of 1:300, following the manufacturer's protocol. Caspase-3 activity was assessed using the CaspGLOW Fluorescein Active Caspase-3 Staining Kit (Catalog No. 88-7004, Thermo Fisher Scientific, Waltham, MA, USA), also at a dilution of 1:300. After staining, the SH-SY5Y cells were washed and resuspended in staining buffer before being subjected to flow cytometric analysis. A representative scatter plot for this analysis is provided in Figures S5 and S6 (Supporting Information).

#### 2.4.5. Quantitation of DNA Fragmentation by TUNEL Assay [38]

DNA fragmentation was evaluated using the Apo-BrdU Apoptosis Detection Kit (Catalog No. A23210, Invitrogen, Thermo Fisher Scientific, Waltham, MA, USA), following the manufacturer's protocol. After treatment, SH-SY5Y cells were washed twice with 1x cold phosphate-buffered saline (PBS), ensuring the collection of both adherent and floating cells. The cell pellets were fixed in 4% paraformaldehyde and 70% ethanol at low temperature, and stored at −20°C for at least 12 h. DNA strand breaks were labeled with BrdU, followed by incubation with the FITC-conjugated anti-BrdU antibody provided in the kit, used at a 1:50 dilution. All incubations were performed in the dark at room temperature for 30 min. Labeled cells were analyzed on a BD Accuri C6 flow cytometer (BD Biosciences, San Jose, CA, USA), acquiring at least 10,000 events per sample. Data analysis was conducted using BD Accuri C6 software. A representative scatter plot for this analysis is provided in Figure S7 (Supporting Information).

#### 2.4.6. Annexin V-Fluorescein Isothiocyanate (FITC) Staining Analysis [39]

Annexin V-FITC/propidium iodide (PI) staining was performed using the FITC Annexin V Apoptosis Detection Kit with PI (Catalog No. 640914, BioLegend, San Diego, CA, USA), according to the manufacturer's instructions. Briefly, single-cell suspensions were incubated at room temperature for 15 min in the dark with annexin V-FITC and PI, each prepared at a 1:20 dilution (v/v). Following staining, the SH-SY5Y cells were washed and resuspended in binding buffer. The apoptotic profile of the cells was analyzed using a BD Accuri C6 flow cytometer (BD Biosciences, San Jose, CA, USA). A representative scatter plot for this analysis is provided in Figure S8 (Supporting Information).

### 2.5. Statistical Analysis

At least three trials were conducted for each experiment. Values represent means ± standard deviation (SD). We performed statistical analysis using Statistical Software for Social Sciences (SPSS). Analyzing the data involved using Duncan's multiple range test and one-way analysis of variance (ANOVA). The threshold for statistical significance was established at a significance level of *p* < 0.05.

## 3. Results and Discussion

Undifferentiated SH-SY5Y cells lack inherent dopaminergic characteristics and are not suitable for directly modeling the pathological mechanisms of dopaminergic neurons or PD. Accordingly, the present study utilizes SH-SY5Y cells as a widely accepted in vitro system, commonly employed in oxidative stress and neuroprotection research, to investigate the cytoprotective potential of RPE against H_2_O_2_-induced apoptosis.

### 3.1. Neuroprotective Activity of RPE in SH-SY5Y Cells

It is widely recognized that neuronal apoptosis plays a pivotal role in both acute and chronic neurodegenerative disorders triggered by metabolic or neurotoxic stressors [41–44].

### 3.2. RPE Preserves Cell Viability Against H_2_O_2_-Induced Cytotoxicity

**Figure 1:**
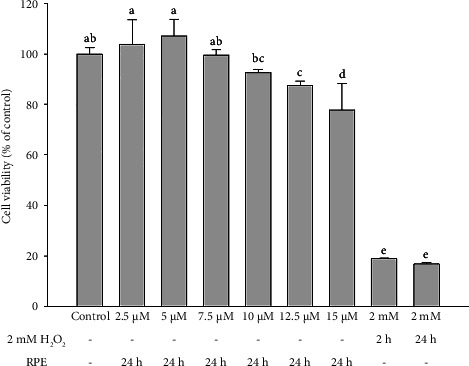
Effect of RPE or H_2_O_2_ treatment on the cell viability of SH-SY5Y cells. SH-SY5Y cells were treated with RPE at concentrations ranging from 0 to 15 μM for 24 h or with 2 mM H_2_O_2_ for 24 h. Cell viability was determined using the MTT assay. Control (CON) represents untreated cells. Values are expressed as the mean ± SD (*n* = 3). Means that share at least one common letter do not differ significantly (*p* < 0.05).

The MTT test was used to assess the viability of SH-SY5Y cells after they were treated with various concentrations of RPE (0–15 μM) or with 2 mM H_2_O_2_ for 24 h, in order to determine the cytotoxic effects of RPE and oxidative stress on SH-SY5Y cells. As demonstrated in Figure 1, at doses ranging from 0 to 7.5 μM, none of the RPE compounds investigated demonstrated cytotoxicity against SH-SY5Y cells. RPE triggered 20% death, however, at 15 μM (80% survival). On the other hand, the survival rate dropped to less than 20% after treatment with 2 mM H_2_O_2_ for both 2 and 24 h. Therefore, for subsequent pretreatment experiments, SH-SY5Y cells were exposed to 2.5 μM or 5 μM RPE followed by 2 mM H_2_O_2_ treatment for 2 h. H_2_O_2_ is a significant generator of reactive oxygen species (ROS), which elicit neuronal destruction through the initiation of apoptosis [45, 46]. To confirm the consistency of the observed viability pattern, we repeated the experiment using LDH release assay, which reflects membrane integrity and cytotoxicity. This observation was further confirmed by LDH release assay, which yielded comparable results to the MTT assay (Supporting Figure S9).

### 3.3. Protection of MMP by RPE

Several assays were conducted using flow cytometry to investigate further the protective effects of RPE against H_2_O_2_-induced neuronal cellular apoptosis. These assays included measuring the MMP, assessing the modulation of Bcl-2 and Bax proteins, examining the release of cytochrome c, evaluating the activation of caspase-9 and caspase-3, analyzing DNA fragmentation, and conducting annexin V-FITC assays. Prior research has shown that ROS can impact mitochondria by opening mitochondrial permeability transition pores (mPTPs) and activating mitochondrial adenosine 5′-triphosphate (ATP)-sensitive potassium (mitoKATP) channels [47]. Cell fatality results from the irreversible initiation of the mPTP, which marks the start of programmed cell death or apoptosis [48]. The preservation of MMP is essential for the generation of ATP and the upkeep of homeostasis in cells [49]. Therefore, the depletion of MMP is a distinguishing feature of apoptosis and is closely linked to its initiation [50–52]. MMP loss was quantified using a potentiometric fluorescent dye known as TMRE. Mitochondria maintain a negative membrane potential relative to the cytoplasm, and because TMRE is positively charged, it is drawn into the mitochondria. The higher the MMP, the more TMRE accumulates within the mitochondria, making it a useful tool for assessing mitochondrial activity and health [53].

**Figure 2:**
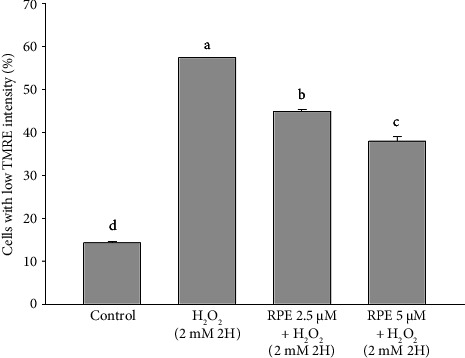
Effects of H_2_O_2_ treatment with or without RPE pretreatment on MMP in SH-SY5Y cells. SH-SY5Y cells were pretreated with RPE at concentrations of 2.5 or 5 μM for 24 h, followed by treatment with 2 mM H_2_O_2_ for 2 h. MMP was then determined by TMRE staining and flow cytometry. Values are expressed as the mean ± SD (*n* = 3). Means that share at least one common letter do not differ significantly (*p* < 0.05).

Fluorescent proteins and other cell components interact with TMRE as it enters a cell, causing the cell to release fluorescence. A cell's fluorescence signal increases as its membrane potential falls and TMRE builds up inside the cell; conversely, when the membrane potential rises, TMRE is discharged from the cell and the fluorescent signal becomes weaker [38]. Consequently, the assessment of TMRE accumulation in mitochondria can serve to evaluate mitochondrial function. The results of subjecting SH-SY5Y cells to 2 mM H_2_O_2_ increased the proportion of low TMRE cells significantly (Figure 2). This increase was equivalent to the decrease in the proportion of high TMRE cells and depletion of MMP (*p* < 0.05). This observation suggests that H_2_O_2_ can induce membrane depolarization, consequently increasing TMRE accumulation.

### 3.4. RPE Modulation of Bcl-2 and Bax by RPE

**Figure 3:**
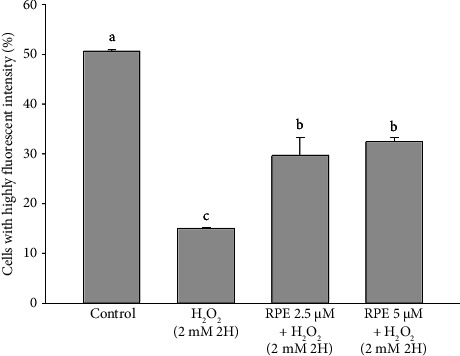
Effects of H_2_O_2_ treatment with or without RPE pretreatment on Bcl-2 expression in SH-SY5Y cells. SH-SY5Y cells were pretreated with RPE at concentrations of 2.5 or 5 μM for 24 h, followed by treatment with 2 mM H_2_O_2_ for 2 h. The level of Bcl-2 expression was determined by flow cytometry. Values are expressed as the mean ± SD (*n* = 3). Means that share at least one common letter do not differ significantly (*p* < 0.05).

The findings of this study provide clear evidence that RPE effectively safeguarded SH-SY5Y cells against mitochondrial dysfunction induced by H_2_O_2_. Bcl-2 belongs to the family of proteins known as B-cell leukemia-2 gene products (Bcl-2), primarily inhibiting apoptosis. Previous studies have proposed that Bcl-2 hinders MMP depolarization, consequently leading to a delay in the activation of different death effectors, including the release of cytochrome c, apoptosis-inducing factor (AIF), and Smac/Diablo [54–56]. On the other hand, Bcl-2 expression suppression results in cellular apoptosis.  Figure 3 shows the findings of a study examining the effect of RPE on Bcl-2 expression in SH-SY5Y cells. The SH-SY5Y cells were subjected to H_2_O_2_ at a concentration of 2 mM for a duration of 2 h. This exposure resulted in a notable and statistically significant reduction in the levels of Bcl-2 when compared to the control cells that were not treated.

**Figure 4:**
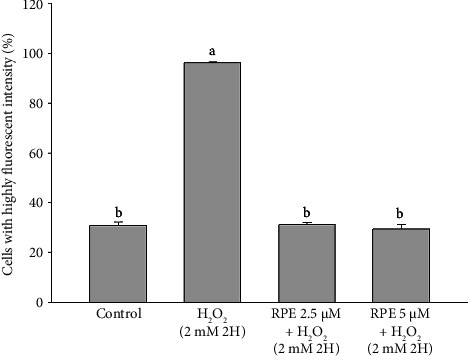
Effects of H_2_O_2_ treatment with or without RPE pretreatment on Bax expression in SH-SY5Y cells. SH-SY5Y cells were pretreated with RPE at concentrations of 2.5 or 5 μM for 24 h, followed by treatment with 2 mM H_2_O_2_ for 2 h. The level of Bax expression was determined by flow cytometry. Values are expressed as the mean ± SD (*n* = 3). Means that share at least one common letter do not differ significantly (*p* < 0.05).

In contrast, a significant increase in Bcl-2 level (*p* < 0.05) was observed when cells were exposed to 2 mM H_2_O_2_ in the presence of either 2.5 or 5 μM RPE. These results demonstrate that RPE rescues SH-SY5Y cells from the H_2_O_2_-induced decrease in Bcl-2 expression. Furthermore, Bax, a pro-apoptotic Bcl-2 protein family constituent, is believed to stimulate various downstream death effectors [57–60].  Figure 4 illustrates the impact of RPE on the expression of Bax in SH-SY5Y cells. The SH-SY5Y cells were subjected to H_2_O_2_ at a concentration of 2 mM for a duration of 2 h. This exposure resulted in a notable and statistically significant elevation in the levels of Bax protein when compared to the control cells that were not treated with H_2_O_2_. In an alternative approach, the cells were subjected to a treatment of 2 mM H_2_O_2_ in the presence of either 2.5 or 5 μM RPE. As a result, a notable decrease in the expression of Bax was observed, indicating a significant impact (*p* < 0.05). The findings of this study indicate that the use of RPE effectively shields SH-SY5Y cells from the detrimental effects of H_2_O_2_-induced elevation in Bax expression.

**Figure 5:**
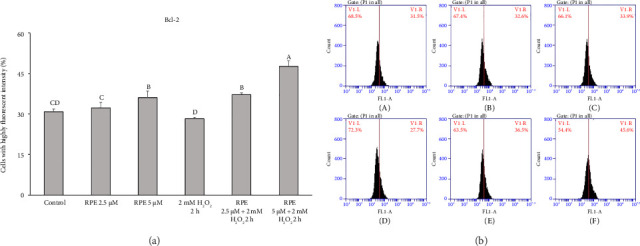
Effects of H_2_O_2_ treatment with or without RPE pretreatment on Bcl-2 expression in SH-SY5Y cells. SH-SY5Y cells were pretreated with RPE at concentrations of 2.5 or 5 μM for 24 h, followed by treatment with 2 mM H_2_O_2_ for 2 h. The level of Bcl-2 expression was determined by flow cytometry. Values are expressed as the mean ± SD (*n* = 3). Means that share at least one common letter do not differ significantly (*p* < 0.05). (A) Control, (B) RPE 2.5 μM, (C) RPE 5 μM, (D) H_2_O_2_, (E) RPE 2.5 μM + H_2_O_2_, and (F) RPE 5 μM + H_2_O_2_.

**Figure 6:**
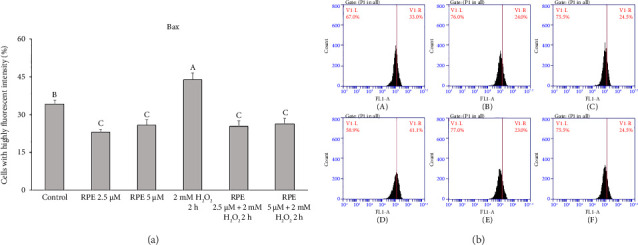
Effects of H_2_O_2_ treatment with or without RPE pretreatment on Bax expression in SH-SY5Y cells. SH-SY5Y cells were pretreated with RPE at concentrations of 2.5 or 5 μM for 24 h, followed by treatment with 2 mM H_2_O_2_ for 2 h. The level of Bax expression was determined by flow cytometry. Values are expressed as the mean ± SD (*n* = 3). Means that share at least one common letter do not differ significantly (*p* < 0.05). (A) Control, (B) RPE 2.5 μM, (C) RPE 5 μM, (D) H_2_O_2_, (E) RPE 2.5 μM + H_2_O_2_, and (F) RPE 5 μM + H_2_O_2_.

To further explore the baseline regulatory effects of RPE on apoptotic signaling under physiological conditions, additional experiments were conducted to assess the expression of Bcl-2 and Bax in cells treated with RPE alone, without H_2_O_2_ exposure. These results, presented in Figures 5 and 6, show that treatment with RPE at 2.5 and 5 μM led to a slight, though not statistically significant, increase in Bcl-2 expression and a corresponding mild reduction in Bax levels relative to the untreated control. While these changes did not reach statistical significance (*p* > 0.05), they suggest a potential intrinsic modulatory effect of RPE on apoptotic homeostasis, even in the absence of oxidative stress. Notably, the most pronounced protective effects were observed in the co-treatment groups, in which RPE significantly reversed H_2_O_2_-induced downregulation of Bcl-2 and upregulation of Bax in a dose-dependent manner. These results underscore the dual role of RPE in both protecting against oxidative damage and maintaining cellular survival signaling under basal conditions.

### 3.5. Inhibition of Cytochrome c Release by RPE

**Figure 7:**
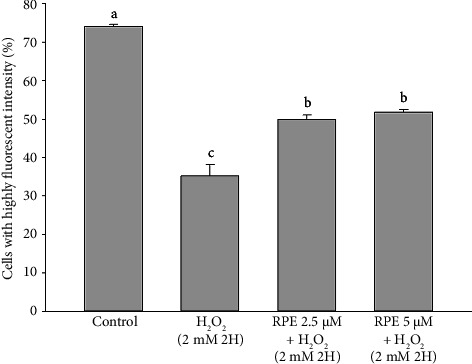
Effects of H_2_O_2_ treatment with or without RPE pretreatment on the release of cytochrome c in SH-SY5Y cells. SH-SY5Y cells were pretreated with RPE at concentrations of 2.5 or 5 μM for 24 h, followed by treatment with 2 mM H_2_O_2_ for 2 h. The level of cytochrome c release was determined by flow cytometry. Values are expressed as the mean ± SD (*n* = 3). Means that share at least one common letter do not differ significantly (*p* < 0.05).

Prior studies have demonstrated that decreased MMP causes the matrix to condense and exposes cytochrome c to the intermembrane space. This process promotes the release of cytochrome c and subsequently triggers apoptotic cell death [61]. The present study investigated the impact of RPE exposure on the release of cytochrome c from SH-SY5Y cells. The population of high fluorescence cells dropped from 74.01% ± 0.66% (control) to 35.20% ± 2.910.53% after the cells were treated with 2 mM H_2_O_2_, suggesting that cytochrome c was released from mitochondria (Figure 7). On the other hand, the populations of high luminous cells increased dramatically to 50.00% ± 1.11% and 51.73% ± 0.70% (*p* < 0.05) when cells were treated with 2.5 or 5 μM RPE. The pathway that relies on the mitochondria involves the liberation of cytochrome c from the intermembrane space of the mitochondria, creating an apoptosome. This apoptosome then triggers the activation of caspase-9 and caspase-3 [62, 63]. However, what is interesting is that the release of cytochrome c from mitochondria treated with H_2_O_2_ may have other mechanisms. For example, Gergalova et al. reported that in H_2_O_2_-treated mitochondria, *α*7 nAChR agonist significantly reduced cytochrome c release by affecting calcium accumulation [64].

### 3.6. Suppression of Caspase Activation by RPE

**Figure 8:**
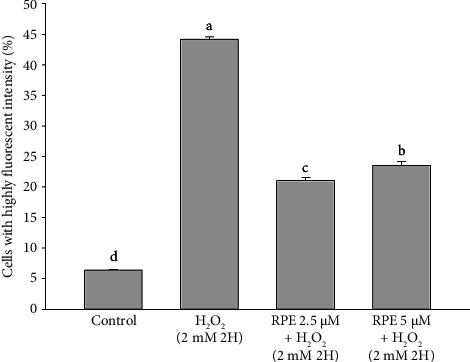
Effects of H_2_O_2_ treatment with or without RPE pretreatment on active caspase-9 expression in SH-SY5Y cells. SH-SY5Y cells were pretreated with RPE at concentrations of 2.5 or 5 μM for 24 h, followed by treatment with 2 mM H_2_O_2_ for 2 h. The level of active caspase-9 expression was determined by flow cytometry. Values are expressed as the mean ± SD (*n* = 3). Means that share at least one common letter do not differ significantly (*p* < 0.05).

**Figure 9:**
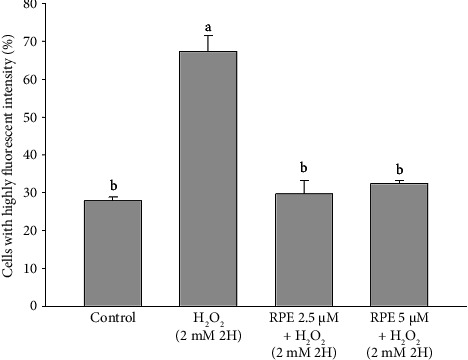
Effects of H_2_O_2_ treatment with or without RPE pretreatment on active caspase-3 expression in SH-SY5Y cells. SH-SY5Y cells were pretreated with RPE at concentrations of 2.5 or 5 μM for 24 h, followed by treatment with 2 mM H_2_O_2_ for 2 h. The level of active caspase-3 expression was determined by flow cytometry. Values are expressed as the mean ± SD (*n* = 3). Means that share at least one common letter do not differ significantly (*p* < 0.05).

The current investigation aimed to assess the impact of RPE on the activation of caspase-9 and caspase-3 in SH-SY5Y cells. The data shown in Figures 8 and 9 show that, compared to the control group, which did not receive any treatment, SH-SY5Y cells exposed to a 2-h exposure to 2 mM H_2_O_2_ had significantly higher levels of active caspase-9 and caspase-3. In contrast, the activation of caspase-9 and caspase-3 was significantly reduced (*p* < 0.05) when cells were exposed to 2 mM H_2_O_2_ in the presence of 2.5 or 5 μM RPE. The findings of this study provide evidence that RPE confers protection to SH-SY5Y cells by inhibiting caspase-9 and caspase-3 activation induced by H_2_O_2_.

### 3.7. RPE Prevents DNA Fragmentation in SH-SY5Y Cells

**Figure 10:**
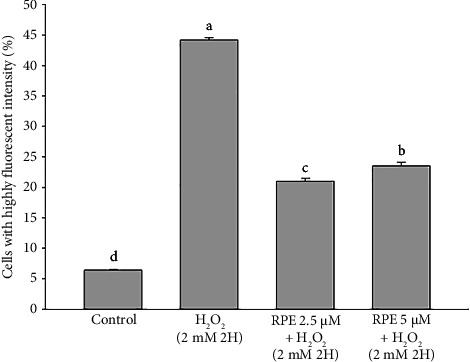
Effects of H_2_O_2_ treatment with or without RPE pretreatment on the extent of DNA fragmentation in SH-SY5Y cells. SH-SY5Y cells were pretreated with RPE at concentrations of 2.5 or 5 μM for 24 h, followed by treatment with 2 mM H_2_O_2_ for 2 h. The extent of DNA fragmentation was determined by flow cytometry. Values are expressed as the mean ± SD (*n* = 3). Means that share at least one common letter do not differ significantly (*p* < 0.05).

The activation of caspase-3 occurs during apoptosis through both the extrinsic and intrinsic pathways. Caspase-3 plays a pivotal role in the initiation of apoptosis and is specifically essential for inducing DNA fragmentation, which results in the distinctive pattern of DNA laddering observed during apoptosis [65, 66]. In order to identify DNA fragmentation, a characteristic feature of advanced apoptosis, a terminal deoxynucleotidyl transferase–mediated dUTP nick end-labeling (TUNEL) assay, was conducted. As illustrated in Figure 10, a notable increase in the population of highly fluorescent cells was observed when the cells were exposed to a concentration of 2 mM H_2_O_2_ for a duration of 2 h. This increase was found to be statistically significant compared to the control group, suggesting the occurrence of DNA fragmentation. On the other hand, it was observed that the population of cells exhibiting high fluorescence levels experienced a notable reduction upon treatment with a concentration of 2 mM H_2_O_2_ while being exposed to either 2.5 or 5 μM RPE. The findings of this study indicate that the use of RPE has the potential to inhibit the initiation of DNA fragmentation in SH-SY5Y cells induced by H_2_O_2_.

### 3.8. Attenuation of Apoptosis and Necrosis by RPE

**Figure 11:**
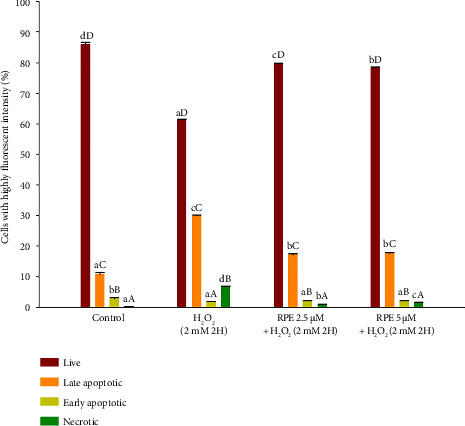
Effects of H_2_O_2_ treatment with or without RPE pretreatment on annexin V-FITC/PI double staining analysis of SH-SY5Y cells. SH-SY5Y cells were pretreated with RPE at concentrations of 2.5 or 5 μM for 24 h, followed by treatment with 2 mM H_2_O_2_ for 2 h. Annexin V-FITC/PI double staining analysis was performed by flow cytometry. The bar graph summarizes the cytometry experiments and displays the percentages of living, early apoptotic, late apoptotic, and necrotic cells according to the treatment, using BD Accuri C6 software analysis. Values are expressed as the mean ± SD (*n* = 3). For each group of bars corresponding to a specific cell population (e.g., live, early apoptotic, late apoptotic, and necrotic), lowercase letters indicate statistical comparisons between different treatment groups, while uppercase letters denote comparisons within the same treatment group across different cell populations. Bars sharing the same letter (uppercase or lowercase) are not significantly different (*p* > 0.05), whereas different letters indicate statistically significant differences (*p* < 0.05).

The early occurrence of apoptosis is associated with the loss of asymmetry in the plasma membrane, leading to the external exposure of phosphatidylserine (PS) residues [67]. The apoptosis detection can be achieved through annexin V, which targets the loss of integrity in the plasma membrane [67]. The observation of a robust and specific interaction between annexin V and PS supports this. Hence, the utilization of annexin V staining represents a feasible approach for the timely identification of apoptosis. The current study analyzed early- and late-stage apoptotic and necrotic cells using annexin V-FITC and PI double staining. The permeability of PI differs between living and nonliving cells, with the membranes of living cells impermeable to PI, while those of dead and damaged cells exhibit permeability. As an illustration, viable cells lack annexin V-FITC and PI staining, while cells in the early stages of apoptosis display positive annexin V-FITC and negative PI staining. The late apoptotic cells exhibited positive staining for annexin V-FITC and PI, while the necrotic cells displayed negative staining for annexin V-FITC and positive staining for PI.  Figure 11 illustrates that the exposure of SH-SY5Y cells to a concentration of 2 mM H_2_O_2_ led to a notable rise in the proportion of cells undergoing late apoptosis and necrosis, while concurrently observing a decline in the percentage of viable cells compared to the control group. Significant reductions in late apoptotic and necrotic cell percentages were observed when cells were exposed to a concentration of 2 mM H_2_O_2_ in the presence of either 2.5 or 5 μM RPE. Additionally, there was a notable increase in the percentage of live cells. The findings of this study provide clear evidence that the use of RPE effectively safeguards SH-SY5Y cells against cell death induced by H_2_O_2_, specifically in terms of apoptosis and necrosis.

### 3.9. Potential Mechanisms and Clinical Implications

Pourhanifeh et al. reported a role in the progression of diseases affecting the central nervous system by utilizing data on the roles of inflammation, oxidative stress, angiogenesis, and apoptosis in neurodegenerative diseases [68]. Potentially mitigating inflammation-induced cell injury is the function of resveratrol. This polyphenol influences cellular processes such as autophagy and apoptotic cascades in response to stress; thus, existing evidence suggests that resveratrol may play a beneficial role in the treatment of neurodegenerative diseases [68].

## 4. Conclusions

This study demonstrates that reactive RPE consistently protected SH-SY5Y cells from apoptosis induced by H_2_O_2_ exposure. The protective effects were evidenced by changes in MMP, modulation of Bcl-2 and Bax protein expression, cytochrome c release, activation of caspase-9 and caspase-3, DNA fragmentation, and increased annexin V-FITC/PI double staining. Although SH-SY5Y cells are widely used as a neuronal-like model, they possess heterogeneous characteristics and do not inherently represent dopaminergic neurons unless differentiated. To further elucidate the mechanisms of RPE action, future studies should utilize differentiated SH-SY5Y cells or in vivo models. Based on the cytoprotective effects observed under oxidative stress, RPE may hold promise as neuroprotective agents in oxidative injury-related neurological disorders.

## Data Availability

The data that support the findings of this study are available upon request from the authors.

## Supporting Information

The supporting information provides detailed flow cytometric dot plots and cytotoxicity assessments supporting the main findings of this study. Figure S1 presents flow cytometric dot plots analyzing the mitochondrial membrane potential (MMP) under various treatment conditions. Figure S2 and Figure S3 display dot plots of the expression levels of antiapoptotic protein Bcl-2 and proapoptotic protein Bax, respectively. Figure S4 illustrates flow cytometric dot plots showing the release of cytochrome c from mitochondria into the cytosol. Figures S5 and S6 show dot plots representing the activation of caspase-9 and caspase-3, respectively, as indicators of the intrinsic apoptotic pathway. Figure S7 provides flow cytometric dot plots confirming DNA fragmentation and apoptotic progression. Figure S8 displays flow cytometric dot plots of annexin V-FITC/PI double staining, used to distinguish live, early apoptotic, and late apoptotic/necrotic SH-SY5Y cells. Figure S9 summarizes MTT assay results reflecting cell viability after RPE or H_2_O_2_ treatment, with statistical significance among groups indicated. These supporting figures support the molecular and cytological mechanisms discussed in the Results and Discussion sections.

## References

[1] Olufunmilayo E. O., Gerke-Duncan M. B., Holsinger R. M. D. (2023). Oxidative Stress and Antioxidants in Neurodegenerative Disorders. *Antioxidants*.

[2] Wakatsuki S., Araki T. (2023). Novel Insights Into the Mechanism of Reactive Oxygen Species-Mediated Neurodegeneration. *Neural Regeneration Research*.

[3] Reed T. T. (2011). Lipid Peroxidation and Neurodegenerative Disease. *Free Radical Biology and Medicine*.

[4] Gu T., Wang N., Wu T., Ge Q., Chen L. (2021). Antioxidative Stress Mechanisms Behind Resveratrol: a Multidimensional Analysis. *Journal of Food Quality*.

[5] Meng T., Xiao D., Muhammed A., Deng J., Chen L., He J. (2021). Anti-Inflammatory Action and Mechanisms of Resveratrol. *Molecules*.

[6] DaCosta D. C. F., Fialho E., Silva J. L. (2017). Cancer Chemoprevention by Resveratrol: The p53 Tumor Suppressor Protein as a Promising Molecular Target. *Molecules*.

[7] Wang Q., Yu Q., Wu M. (2022). Antioxidant and Neuroprotective Actions of Resveratrol in Cerebrovascular Diseases. *Frontiers in Pharmacology*.

[8] Carpéné C., Les F., Cásedas G. (2019). Resveratrol Anti-Obesity Effects: Rapid Inhibition of Adipocyte Glucose Utilization. *Antioxidants*.

[9] Li J., Zhang C. X., Liu Y. M., Chen K. L., Chen G. (2017). A Comparative Study of Anti-Aging Properties and Mechanism: Resveratrol and Caloric Restriction. *Oncotarget*.

[10] Cosar M. Y., Erdogan M. A., Yilmaz O. (2023). Epigallocatechin-3-Gallate and Resveratrol Attenuate Hydrogen Peroxide Induced Damage in Neuronal Cells. *Bratislava Medical Journal*.

[11] Cracco P., Montalesi E., Parente M. (2023). A Novel Resveratrol-Induced Pathway Increases Neuron-Derived Cell Resilience Against Oxidative Stress. *International Journal of Molecular Sciences*.

[12] Akyuva Y., Nazıroğlu M. (2020). Resveratrol Attenuates Hypoxia-Induced Neuronal Cell Death, Inflammation and Mitochondrial Oxidative Stress by Modulation of TRPM2 Channel. *Scientific Reports*.

[13] Wu P. F., Xie N., Zhang J. J. (2013). Resveratrol Preconditioning Increases Methionine Sulfoxide Reductases A Expression and Enhances Resistance of Human Neuroblastoma Cells to Neurotoxins. *The Journal of Nutritional Biochemistry*.

[14] González-Sarrías A., Núñez-Sánchez M. Á., Tomás-Barberán F. A., Espín J. C. (2017). Neuroprotective Effects of Bioavailable Polyphenol-Derived Metabolites Against Oxidative Stress-Induced Cytotoxicity in Human Neuroblastoma SH-SY5Y Cells. *Journal of Agricultural and Food Chemistry*.

[15] Penãlver P., Zodio S., Lucas R., De-Paz M. V., Morales J. C. (2020). Neuroprotective and Anti-Inflammatory Effects of Pterostilbene Metabolites in Human Neuroblastoma SH-SY5Y and RAW 264.7 Macrophage Cells. *Journal of Agricultural and Food Chemistry*.

[16] Moon H. R., Yun J. M. (2023). Neuroprotective Effects of Hesperetin on H_2_O_2_-Induced Damage in Neuroblastoma SH-SY5Y Cells. *Nutrition Research and Practice*.

[17] Singh A., Kukreti R., Saso L., Kukreti S. (2019). Oxidative Stress: A Key Modulator in Neurodegenerative Diseases. *Molecules*.

[18] Kung H. C., Lin K. J., Kung C. T., Lin T. K. (2021). Oxidative Stress, Mitochondrial Dysfunction, and Neuroprotection of Polyphenols with Respect to Resveratrol in Parkinson’s Disease. *Biomed*.

[19] Prakash S., Carter W. G. (2021). The Neuroprotective Effects of Cannabis-Derived Phytocannabinoids and Resveratrol in Parkinson’s Disease: A Systematic Literature Review of Pre-Clinical Studies. *Brain Sciences*.

[20] Wang Z. H., Zhang J. L., Duan Y. L., Zhang Q. S., Li G. F., Zheng D. L. (2015). MicroRNA-214 Participates in the Neuroprotective Effect of Resveratrol via Inhibiting α-Synuclein Expression in MPTP-Induced Parkinson’s Disease Mouse. *Biomedicine & Pharmacotherapy*.

[21] Rahman M. H., Akter R., Bhattacharya T. (2020). Resveratrol and Neuroprotection: Impact and Its Therapeutic Potential in Alzheimer’s Disease. *Frontiers in Pharmacology*.

[22] Yang A. J. T., Bagit A., Macpherson R. E. K. (2021). Resveratrol, Metabolic Dysregulation, and Alzheimer’s Disease: Considerations for Neurogenerative Disease. *International Journal of Molecular Sciences*.

[23] Anekonda T. S. (2006). Resveratrol—A Boon for Treating Alzheimer’s Disease?. *Brain Research Reviews*.

[24] Amri A., Chaumeil J. C., Sfar S., Charrueau C. (2012). Administration of Resveratrol: What Formulation Solutions to Bioavailability Limitations?. *Journal of Controlled Release*.

[25] Walle T. (2011). Bioavailability of Resveratrol. *Annals of the New York Academy of Sciences*.

[26] Vesely O., Baldovska S., Kolesarova A. (2021). Enhancing Bioavailability of Nutraceutically Used Resveratrol and Other Stilbenoids. *Nutrition*.

[27] DeVries K., Strydom M., Steenkamp V., Netticadan T., Wigle J., Raj P. (2021). A Brief Updated Review of Advances to Enhance Resveratrol’s Bioavailability. *Molecules*.

[28] Neises B., Steglich W. (1978). Simple Method for the Esterification of Carboxylic Acids. *Angewandte Chemie International Edition in English*.

[29] Tain Y.-L., Chang S. K. C., Liao J.-X. (2021). Synthesis of Short-Chain-Fatty-Acid Resveratrol Esters and Their Antioxidant Properties. *Antioxidants*.

[30] Hou C. Y., Chen Y. W., Hazeena S. H. (2024). Cardiovascular Risk of Dietary Trimethylamine Oxide Precursors and the Therapeutic Potential of Resveratrol and Its Derivatives. *FEBS Open Bio*.

[31] Tain Y.-L., Jheng L.-C., Chang S. K. C. (2020). Synthesis and Characterization of Novel Resveratrol Butyrate Esters That Have the Ability to Prevent Fat Accumulation in a Liver Cell Culture Model. *Molecules*.

[32] Liao J.-X., Chen Y.-W., Shih M.-K. (2021). Resveratrol Butyrate Esters Inhibit bpa‐Induced Liver Damage in Male Offspring Rats by Modulating Antioxidant Capacity and Gut Microbiota. *International Journal of Molecular Sciences*.

[33] Shih M.-K., Tain Y.-L., Chen Y.-W. (2021). Resveratrol Butyrate Esters Inhibit Obesity Caused by Perinatal Exposure to Bisphenol a in Female Offspring Rats. *Molecules*.

[34] Shih M.-K., Tain Y.-L., Cheng C.-M. (2021). Separation and Identification of Resveratrol Butyrate Ester Complexes and Their Bioactivity in HepG2 Cell Models. *International Journal of Molecular Sciences*.

[35] Arbo B. D., André-Miral C., Nasre-Nasser R. G. (2020). Resveratrol Derivatives as Potential Treatments for Alzheimer’s and Parkinson’s Disease. *Frontiers in Aging Neuroscience*.

[36] Xicoy H., Wieringa B., Martens G. J. M. (2017). The SH-SY5Y Cell Line in Parkinson’s Disease Research: A Systematic Review. *Molecular Neurodegeneration*.

[37] Wang C. Y., Wu T. C., Hsieh S. L., Tsai Y. H., Yeh C. W., Huang C. Y. (2015). Antioxidant Activity and Growth Inhibition of Human Colon Cancer Cells by Crude and Purified Fucoidan Preparations Extracted from Sargassum cristaefolium. *Journal of Food and Drug Analysis*.

[38] Huang C. Y., Kuo C. H., Chen P. W. (2017). Compressional-Puffing Pretreatment Enhances Neuroprotective Effects of Fucoidans From the Brown Seaweed Sargassum Hemiphyllum on 6-Hydroxydopamine-Induced Apoptosis in SH-SY5Y Cells. *Molecules*.

[39] Yang W. N., Chen P. W., Huang C. Y. (2017). Compositional Characteristics and In Vitro Evaluations of Antioxidant and Neuroprotective Properties of Crude Extracts of Fucoidan Prepared from Compressional Puffing-Pretreated Sargassum Crassifolium. *Marine Drugs*.

[40] Yokoyama T., Tanahashi M., Kobayashi Y. (2002). The Expression of Bcl-2 Family Proteins (Bcl-2, Bcl-x, Bax, Bak and Bim) in Human Lymphocytes. *Immunology Letters*.

[41] Shiao W. C., Kuo C. H., Tsai Y. H. (2020). In Vitro Evaluation of Anti-Colon Cancer Potential of Crude Extracts of Fucoidan Obtained From Sargassum glaucescens Pretreated by Compressional-Puffing. *Applied Sciences*.

[42] Andreone B. J., Larhammar M., Lewcock J. W. (2020). Cell Death and Neurodegeneration. *Cold Spring Harbor Perspectives in Biology*.

[43] Block M. L., Hong J. S. (2005). Microglia and Inflammation-Mediated Neurodegeneration: Multiple Triggers With a Common Mechanism. *Progress in Neurobiology*.

[44] Mandemakers W., Morais V. A., DeStrooper B. (2007). A Cell Biological Perspective on Mitochondrial Dysfunction in Parkinson Disease and Other Neurodegenerative Diseases. *Journal of Cell Science*.

[45] Hosseinzadeh L., Malekshahi A., Ahmadi F., Emami S., Hajialyani M., Mojarrab M. (2018). The Protective Effect of Different Extracts of Three Artemisia Species Against H_2_O_2_-Induced Oxidative Stress and Apoptosis in PC12 Neuronal Cells. *Pharmacognosy Research*.

[46] Chen L., Liu L., Yin J., Luo Y., Huang S. (2009). Hydrogen Peroxide-Induced Neuronal Apoptosis Is Associated With Inhibition of Protein Phosphatase 2A and 5, Leading to Activation of MAPK Pathway. *The International Journal of Biochemistry & Cell Biology*.

[47] Kent A. C., ElBaradie K. B. Y., Hamrick M. W. (2021). Targeting the Mitochondrial Permeability Transition Pore to Prevent Age-Associated Cell Damage and Neurodegeneration. *Oxidative Medicine and Cellular Longevity*.

[48] Crompton M. (1999). The Mitochondrial Permeability Transition Pore and Its Role in Cell Death. *Biochemical Journal*.

[49] Tang X. Q., Feng J. Q., Chen J. (2005). Protection of Oxidative Preconditioning Against Apoptosis Induced by H2O2 in PC12 Cells: Mechanisms via MMP, ROS, and Bcl-2. *Brain Research*.

[50] Dadsena S., Jenner A., García-Sáez A. J. (2021). Mitochondrial Outer Membrane Permeabilization at the Single Molecule Level. *Cellular and Molecular Life Sciences*.

[51] Kroemer G., Galluzzi L., Brenner C. (2007). Mitochondrial Membrane Permeabilization in Cell Death. *Physiological Reviews*.

[52] Green D. R., Kroemer G. (2004). The Pathophysiology of Mitochondrial Cell Death. *Science*.

[53] Gan Z., Audi S. H., Bongard R. D., Gauthier K. M., Merker M. P. (2011). Quantifying Mitochondrial and Plasma Membrane Potentials in Intact Pulmonary Arterial Endothelial Cells Based on Extracellular Disposition of Rhodamine Dyes. *American Journal of Physiology-Lung Cellular and Molecular Physiology*.

[54] Mitra S., Rauf A., Sutradhar H. (2023). Potential Candidates from Marine and Terrestrial Resources Targeting Mitochondrial Inhibition: Insights From the Molecular Approach. *Comparative Biochemistry and Physiology, Part C: Toxicology & Pharmacology*.

[55] Penninger J. M., Kroemer G. (2003). Mitochondria, AIF and Caspases--Rivaling for Cell Death Execution. *Nature Cell Biology*.

[56] Antonsson B. (2004). Mitochondria and the Bcl-2 Family Proteins in Apoptosis Signaling Pathways. *Molecular and Cellular Biochemistry*.

[57] Roy M. J., Vom A., Czabotar P. E., Lessene G. (2014). Cell Death and the Mitochondria: Therapeutic Targeting of the BCL-2Family-Driven Pathway. *British Journal of Pharmacology*.

[58] VanDelft M. F., Huang D. C. S. (2006). How the Bcl-2 Family of Proteins Interact to Regulate Apoptosis. *Cell Research*.

[59] Kuwana T., Newmeyer D. D. (2003). Bcl-2-family Proteins and the Role of Mitochondria in Apoptosis. *Current Opinion in Cell Biology*.

[60] Akl H., Vervloessem T., Kiviluoto S. (2014). A Dual Role for the Anti-Apoptotic Bcl-2 Protein in Cancer: Mitochondria Versus Endoplasmic Reticulum. *Biochimica et Biophysica Acta (BBA)-Molecular Cell Research*.

[61] Gottlieb E., Armour S. M., Harris M. H., Thompson C. B. (2003). Mitochondrial Membrane Potential Regulates Matrix Configuration and Cytochrome C Release During Apoptosis. *Cell Death & Differentiation*.

[62] Nguyen T. T. M., Gillet G., Popgeorgiev N. (2021). Caspases in the Developing Central Nervous System: Apoptosis and Beyond. *Frontiers in Cell and Developmental Biology*.

[63] Jiang X., Wang X. (2004). Cytochrome C-Mediated Apoptosis. *Annual Review of Biochemistry*.

[64] Gergalova G., Lykhmus O., Kalashnyk O. (2012). Mitochondria Express α7 Nicotinic Acetylcholine Receptors to Regulate Ca^2+^ Accumulation and Cytochrome C Release: Study on Isolated Mitochondria. *PLoS One*.

[65] Enari M., Sakahira H., Yokoyama H., Okawa K., Iwamatsu A., Nagata S. (1998). A Caspase-Activated DNase That Degrades DNA During Apoptosis, and Its Inhibitor ICAD. *Nature*.

[66] Liu X., Li P., Widlak P. (1998). The 40-kDa Subunit of DNA Fragmentation Factor Induces DNA Fragmentation and Chromatin Condensation During Apoptosis. *Proceedings of the National Academy of Sciences of the United States of America*.

[67] VanEngeland M., Nieland L. J. W., Ramaekers F. C. S., Schutte B., Reutelingsperger C. P. M. (1998). Annexin V-Affinity Assay: A Review on an Apoptosis Detection System Based on Phosphatidylserine Exposure. *Cytometry*.

[68] Pourhanifeh M. H., Shafabakhsh R., Reiter R. J., Asemi Z. (2019). The Effect of Resveratrol on Neurodegenerative Disorders: Possible Protective Actions Against Autophagy, Apoptosis, Inflammation and Oxidative Stress. *Current Pharmaceutical Design*.

