# Supplementary material for: Neuroprotective Effect of Resveratrol Propionate Esters on Apoptosis of SH-SY5Y Cells Induced by Hydrogen Peroxide
Source: Biochem Res Int. 2025 Sep 10;2025:9973711. doi: 10.1155/bri/9973711 (PMC12443518; doi:10.1155/bri/9973711)
Supplement: Supporting Information — Additional supporting information can be found online in the Supporting Information section. [file 9973711.f1.zip › Accepted revised Supplementary Materials Figures.pdf]

Supplementary Materials- Flow cytometric dot plots and LDH activity assays.

Figure S1. MMP analysis.

Figure S2. Bcl-2 analysis.

Figure S3. Bax analysis.

Figure S4. Release of cytochrome c analysis.

Figure S5. Caspase-9.

Figure S6. Caspase-3.

Figure S7. DNA fragmentation.

Figure S8. Annexin V-FITC/PI double staining analysis.

Figure S9. Effect of RPE or H_2O_2 treatment on the cell viability of SH-SY5Y cells. SH-SY5Y cells were treated with RPE at concentrations of 0–15 μM for 24 hours or with 2 mM H_2O_2 for 24 hours. Cell viability was determined using the MTT assay. Control (CON) represents untreated cells. Values are expressed as mean ± SD (n = 3). The means that have at least one common letter do not differ significantly ($p < 0.05$).

Figure S1. MMP analysis.

Control	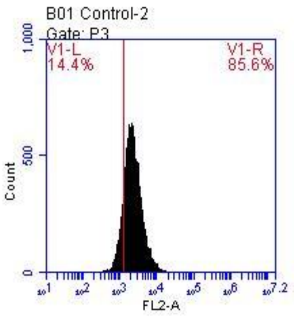	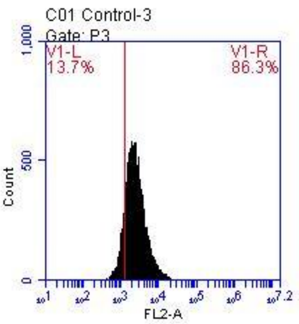	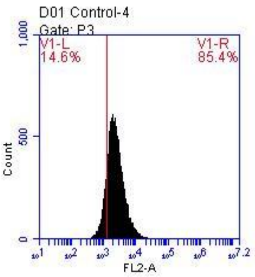
H2O2(2mM)	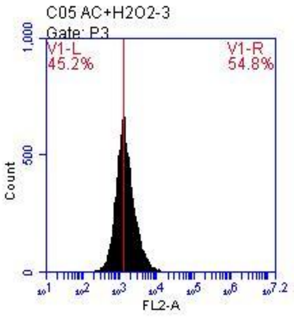	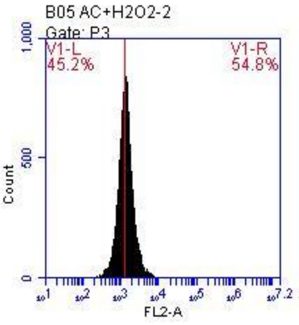	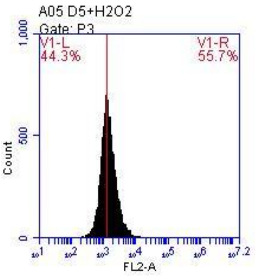
RPE 2.5uM<br>+H2O2(2mM)	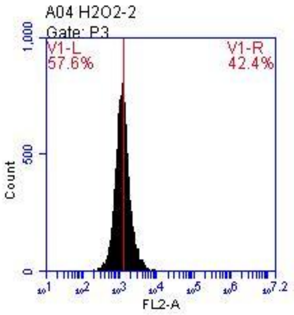	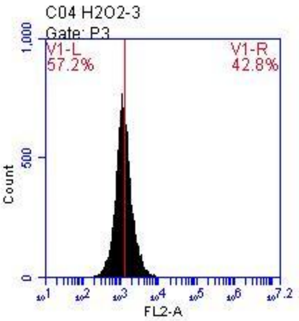	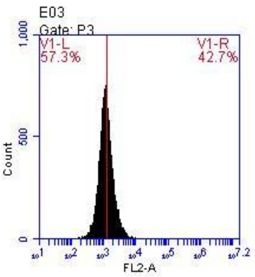
RPE 5uM<br>+H2O2(2mM)	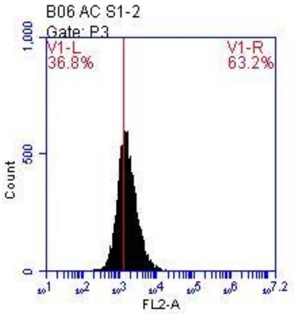	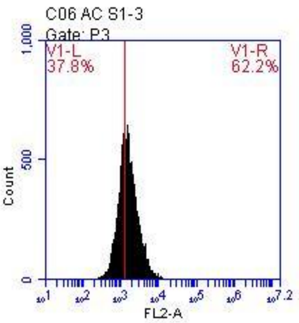	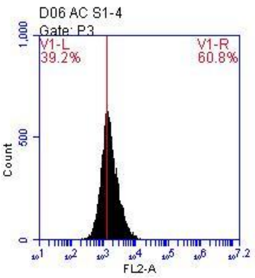

Figure S2. Bcl-2 analysis.

Control	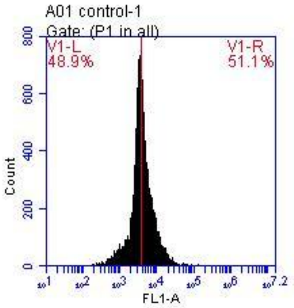	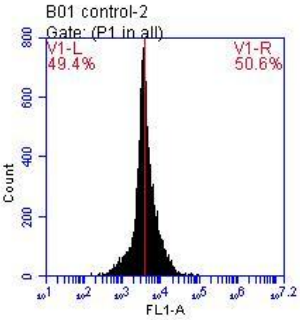	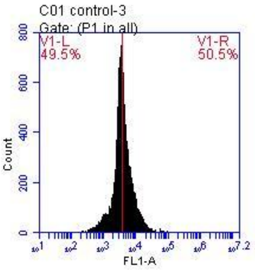
H2O2(2mM)	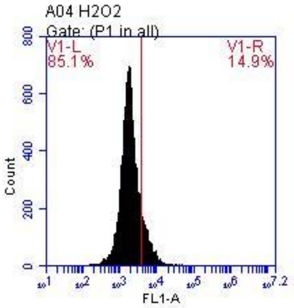	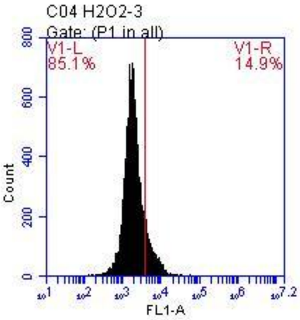	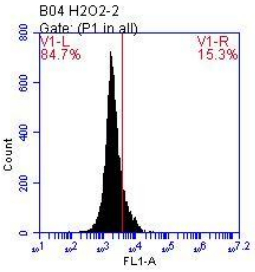
RPE 2.5uM +H2O2(2mM)	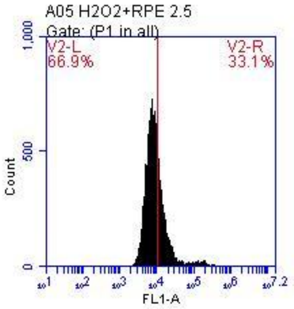	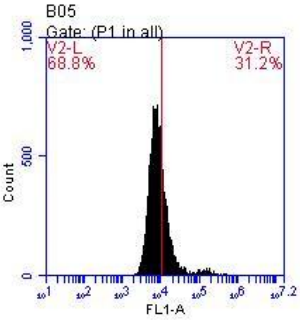	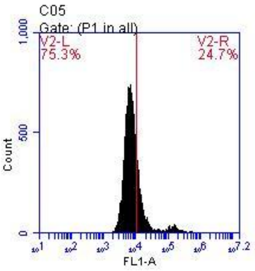
RPE 5uM +H2O2(2mM)	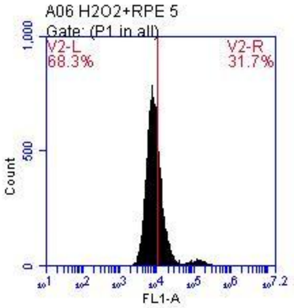	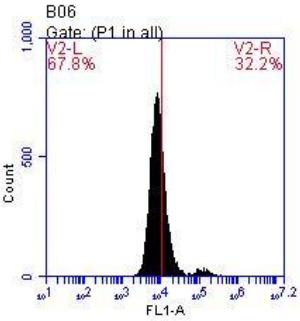	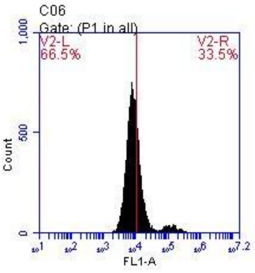

Figure S3. Bax analysis.

Control

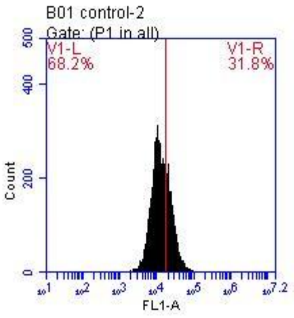

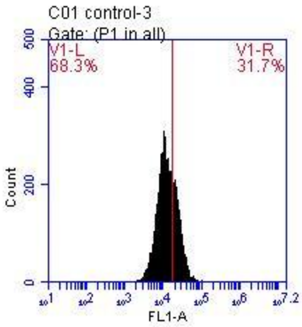

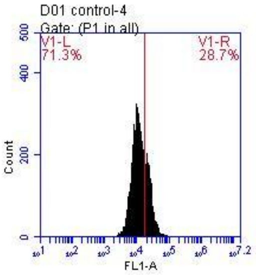

H2O2(2mM)

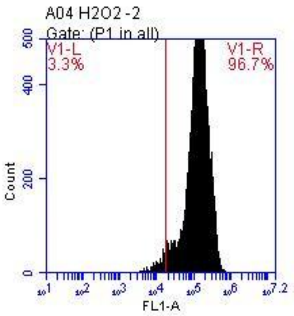

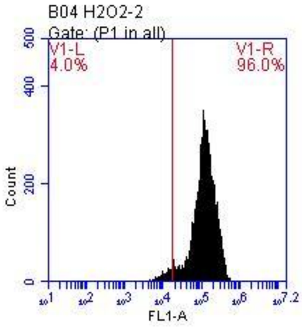

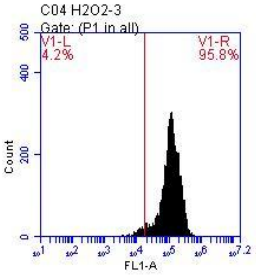

RPE 2.5uM

+H2O2(2mM)

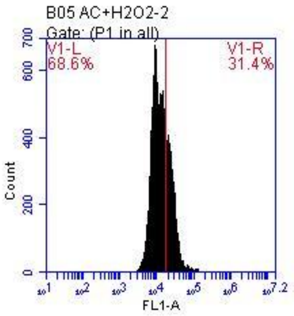

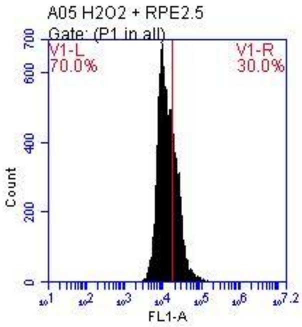

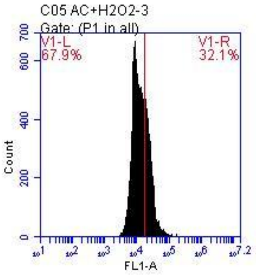

RPE 5uM

+H2O2(2mM)

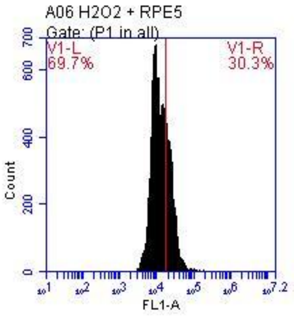

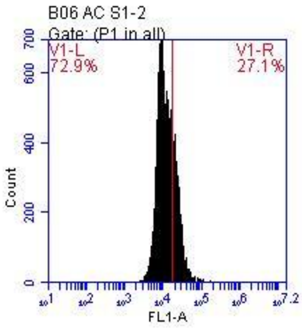

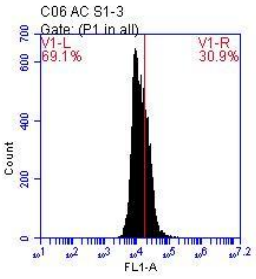

Figure S4. Release of cytochrome c analysis.

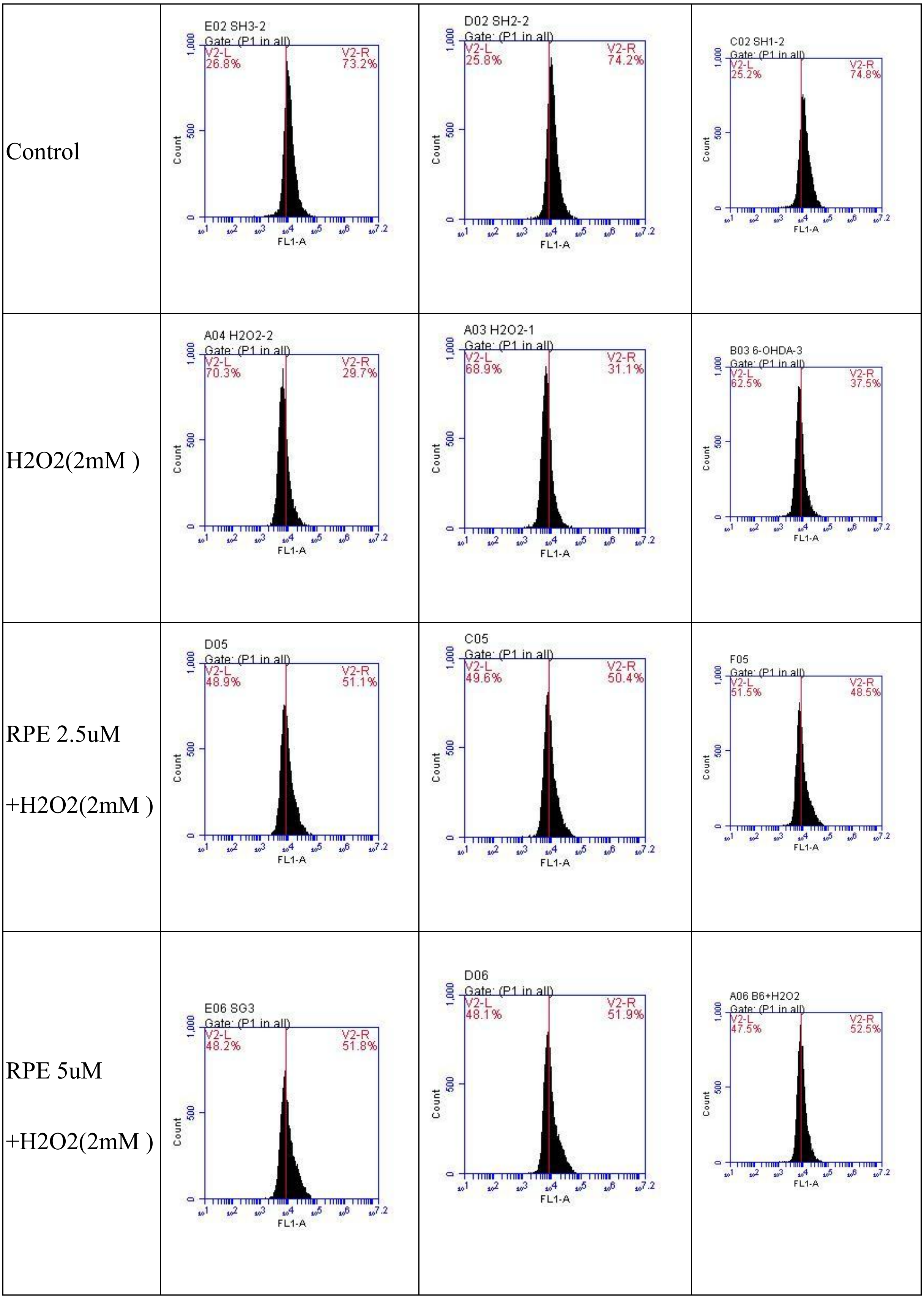

Figure S5. Caspase-9.

<table>
<tr>
<td>Control</td>
<td>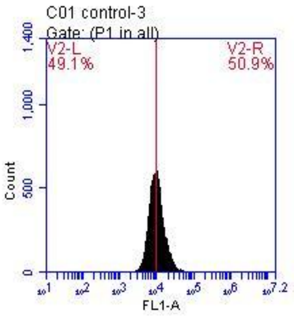
</td>
<td>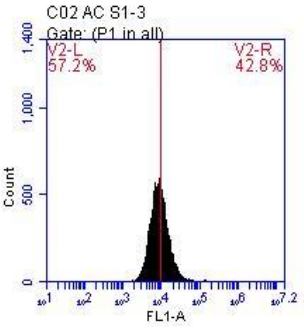
</td>
<td>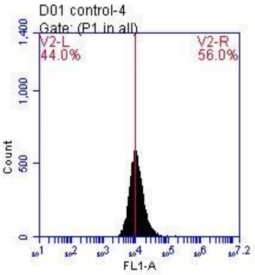
</td>
</tr>
<tr>
<td>H2O2(2mM)</td>
<td>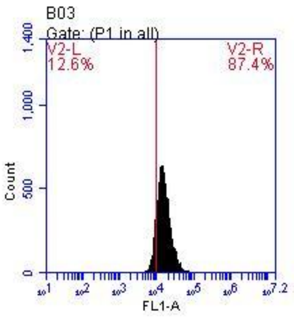
</td>
<td>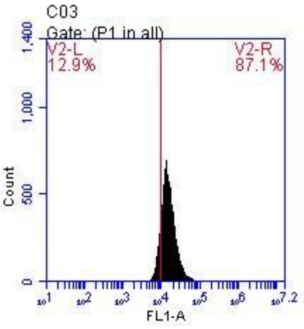
</td>
<td>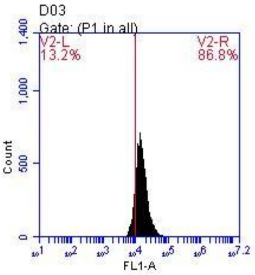
</td>
</tr>
<tr>
<td>RPE 2.5uM
+H2O2(2mM)</td>
<td>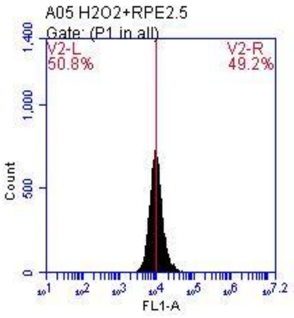
</td>
<td>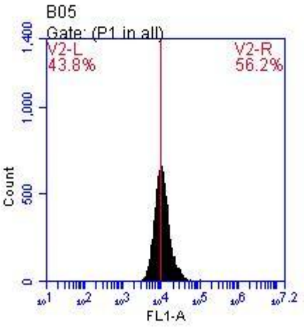
</td>
<td>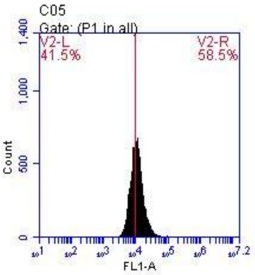
</td>
</tr>
<tr>
<td>RPE 5uM
+H2O2(2mM)</td>
<td>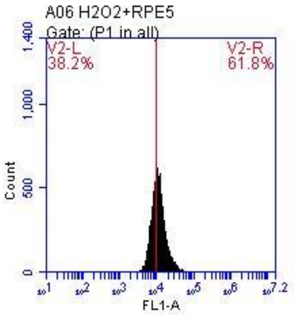
</td>
<td>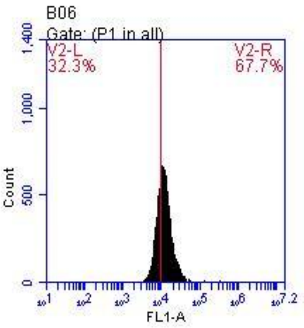
</td>
<td>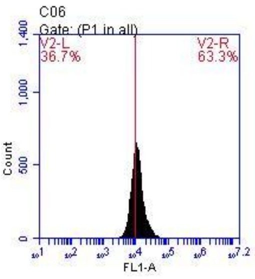
</td>
</tr>
</table>

Figure S6. Caspase-3.

Control

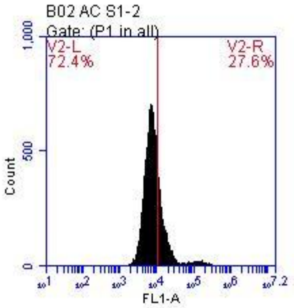

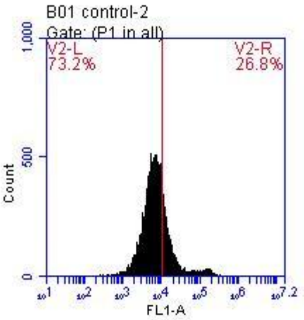

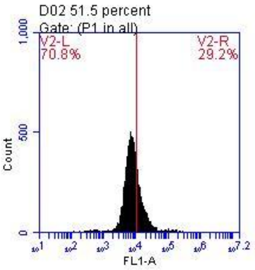

H2O2(2mM)

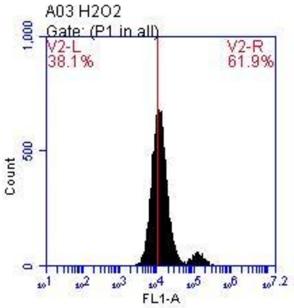

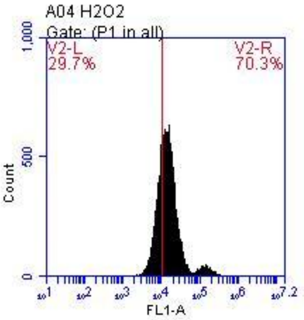

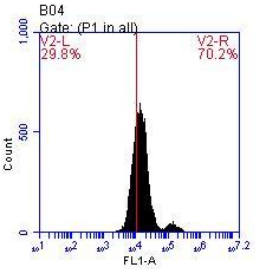

RPE 2.5uM

+H2O2(2mM)

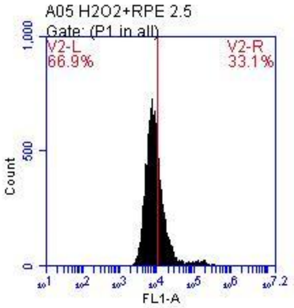

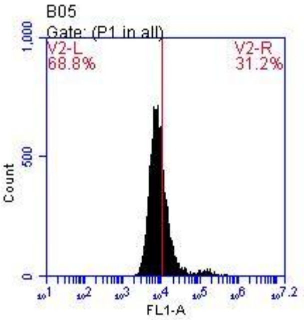

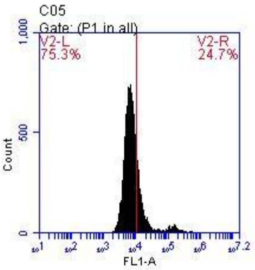

RPE 5uM

+H2O2(2mM)

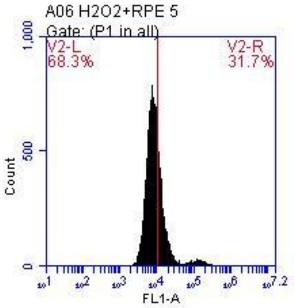

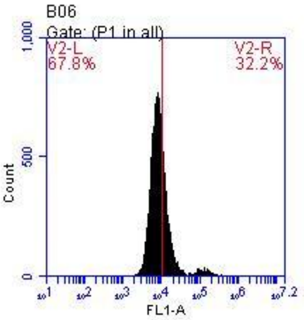

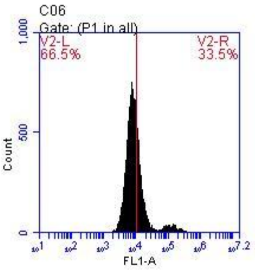

Figure S7. DNA fragmentation.

Control

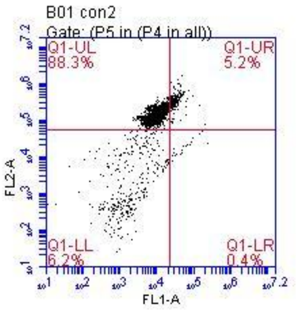

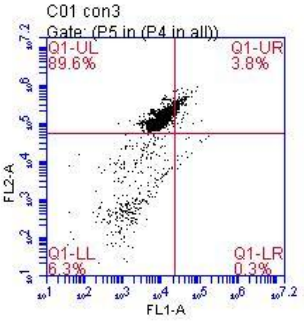

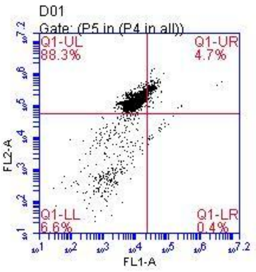

H2O2(2mM)

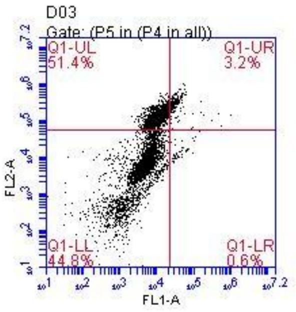

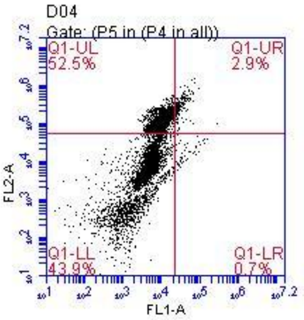

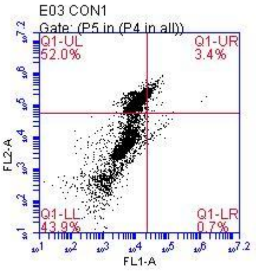

RPE 2.5uM

+H2O2(2mM)

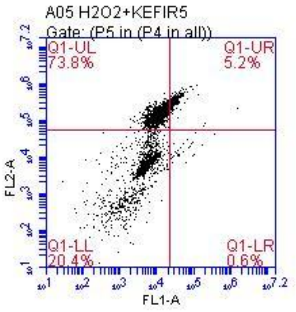

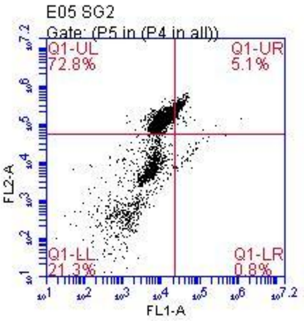

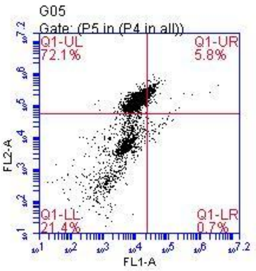

RPE 5uM

+H2O2(2mM)

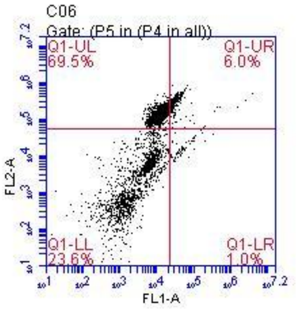

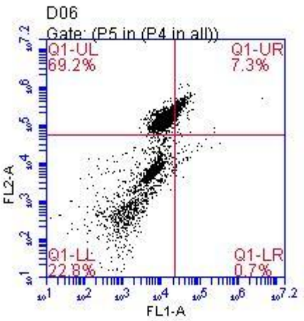

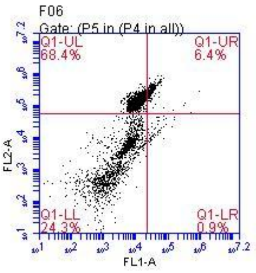

Figure S8. Annexin V-FITC/PI double staining analysis.

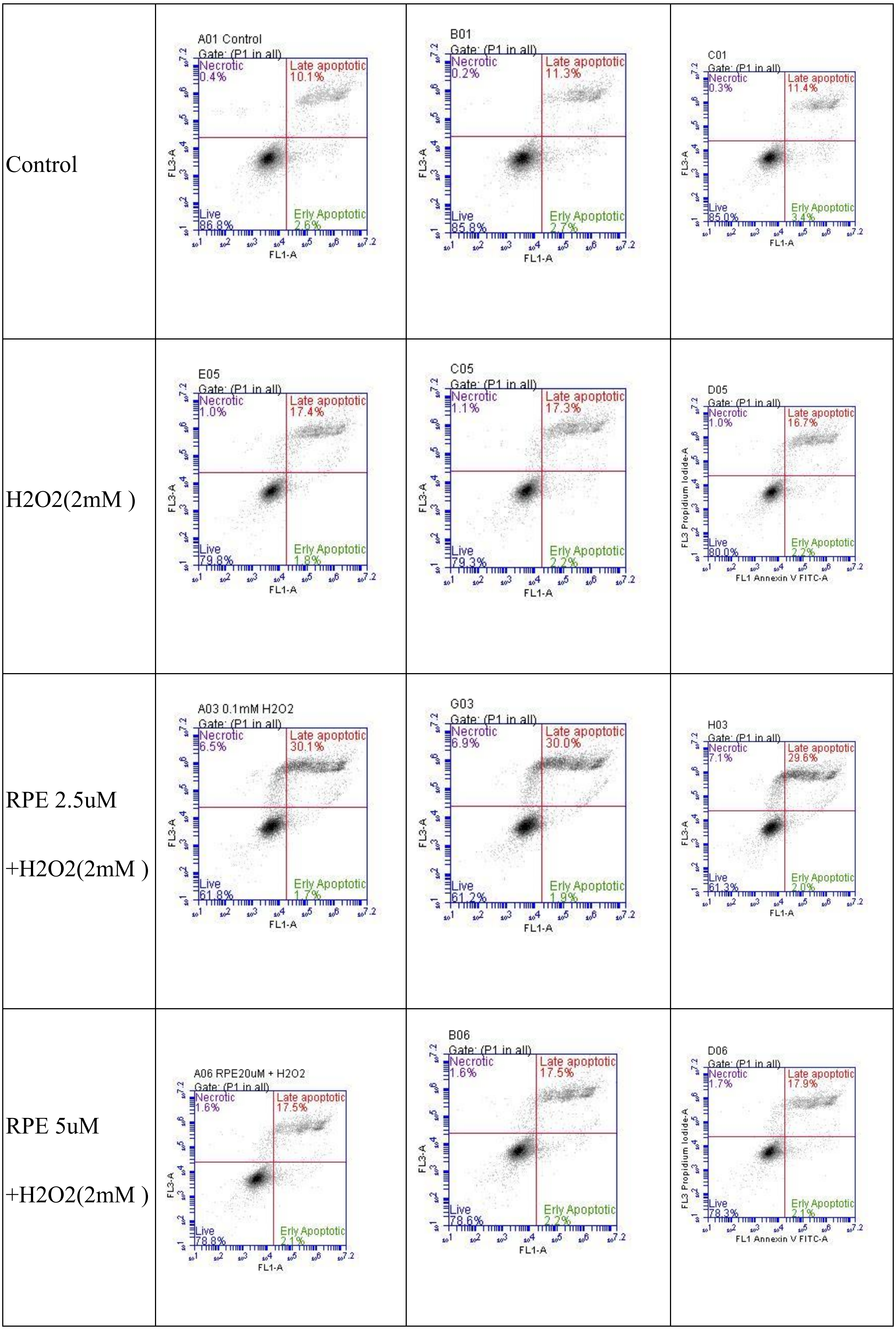

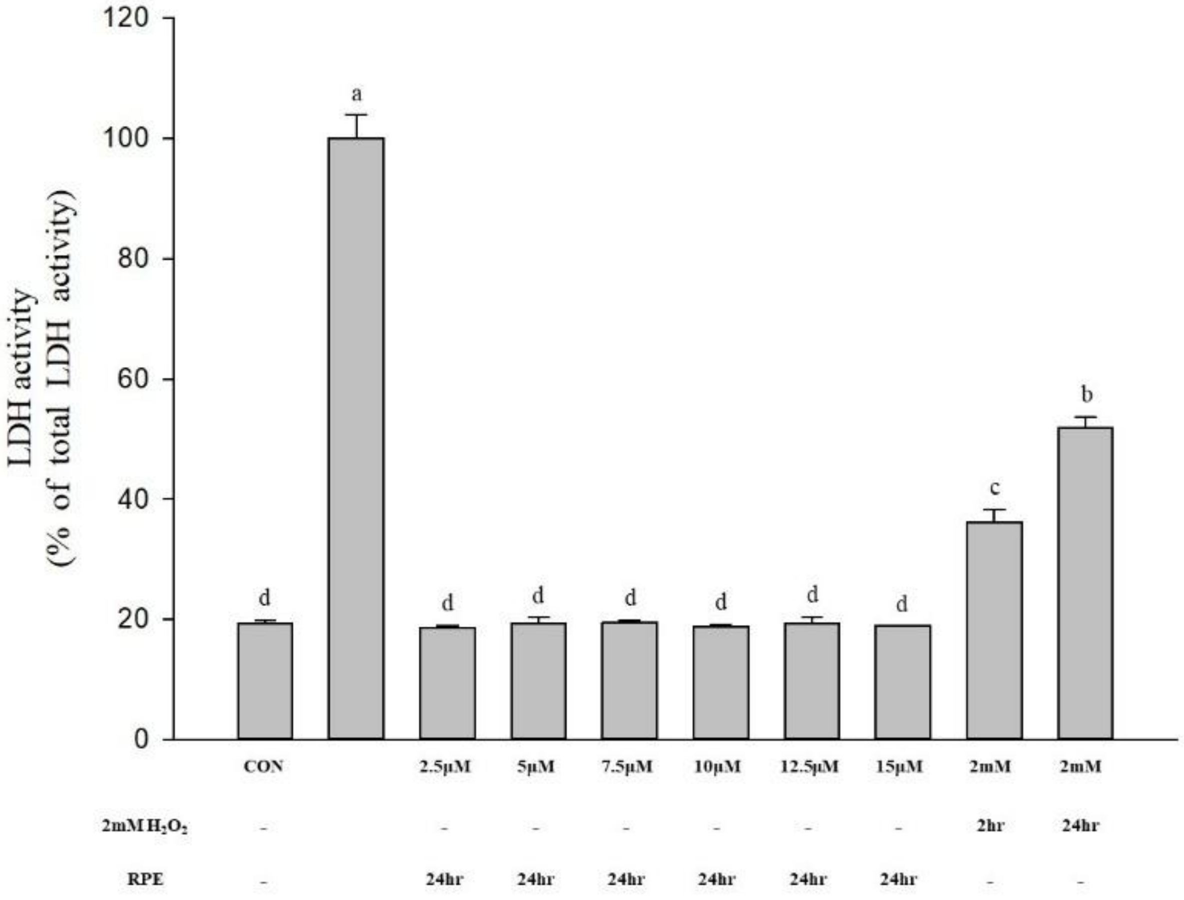

Figure S9. Effect of RPE or H_2O_2 treatment on the cell viability of SH-SY5Y cells. SH-SY5Y cells were treated with RPE at concentrations of 0–15 μM for 24 hours or with 2 mM H_2O_2 for 24 hours. Cell viability was determined using the MTT assay. Control (CON) represents untreated cells. Values are expressed as mean ± SD (n = 3). The means that have at least one common letter do not differ significantly ($p < 0.05$).
